# Successful endoscopic mechanical lithotripsy for post-ERCP gallstone ileus: a case report

**DOI:** 10.3389/fmed.2025.1604835

**Published:** 2025-07-02

**Authors:** Jing Wang, Zhe Luan, Bin Yan, Gang Sun

**Affiliations:** ^1^Department of Gastroenterology and Hepatology, First Medical Center, Chinese PLA General Hospital, Beijing, China; ^2^School of Medicine, Nankai University, Tianjin, China

**Keywords:** gallstone ileus, mechanical lithotripsy, endoscopic retrograde cholangiopancreatography, endoscopic treatment, case report

## Abstract

Gallstone ileus is a rare but severe complication of gallstone disease and is typically caused by the formation of a fistula between the biliary and gastrointestinal systems. The conventional treatment approach is enterolithotomy with stone removal. However, for elderly patients or those with underlying comorbidities, surgical intervention poses significant risks. Therefore, exploring minimally invasive, nonsurgical treatment strategies holds critical clinical value. A 63-year-old female was admitted to the hospital due to abdominal pain, chills, and high fever. She was diagnosed with a large common bile duct stone (diameter 3.9 cm). During endoscopic retrograde cholangiopancreatography (ERCP), as the stone was too large to be removed directly, endoscopic sphincterotomy (EST) combined with the placement of a biliary plastic stent was performed. On the 7th postoperative day, the patient developed nausea, vomiting, and constipation. Laboratory tests revealed elevated inflammatory marker, pancreatic enzyme, bile enzyme, and bilirubin levels. Imaging studies revealed intestinal dilatation and a high-density shadow within the intestinal lumen, leading to the diagnosis of gallstone ileus. On postoperative day 11, biliary and pancreatic stents were placed. On postoperative day 16, endoscopic stone extraction was attempted, successfully relieving the obstruction. The patient recovered well postoperatively and experienced no recurrence during the 2-month follow-up. This case report describes the successful treatment of post-ERCP gallstone ileus using endoscopic stone extraction and fragmentation techniques.

## 1 Introduction

Gallstone ileus is a rare complication of gallstone disease. Its primary pathological mechanism involves chronic inflammation of the biliary tract, which leads to adhesions between the biliary and intestinal systems, resulting in pressure necrosis and the formation of a fistula, most commonly between the gallbladder and the duodenum. This pathological channel allows gallstones to enter the intestinal tract, causing mechanical bowel obstruction (1). The imaging findings typically exhibit the classic Rigler triad: ectopic gallstones, bowel obstruction, and pneumobilia (2). In rare cases, gallstones may pass directly into the duodenum through the common bile duct and an enlarged ampulla of Vater following ERCP and sphincterotomy, leading to bowel obstruction (3–6).

Although the incidence of gallstone ileus is low [accounting for less than 0.1% of cases of mechanical bowel obstruction (7)], its mortality rate is 5–10 times higher than that of other nonmalignant mechanical small bowel obstructions, with reported mortality rates ranging from 12 to 18% (8). It is a surgical emergency requiring significant attention (9). Common risk factors include female sex, age >65 years, a history of untreated cholecystitis, and large gallstones (>2.5 cm). Enterolithotomy is the current mainstay of treatment (10). However, the high risks and complication rates associated with surgical intervention (11) make it unsuitable for elderly patients with poor baseline health. In this context, exploring nonsurgical treatment approaches is critically important.

We report a case in which endoscopic stone extraction combined with mechanical lithotripsy was successfully used to treat ileal gallstone obstruction, providing valuable insights into the management of such patients.

## 2 Case presentation

A 63-year-old female (BMI = 15.79) presented to the hospital with a 3-day history of chills, high fever, and abdominal pain. Ultrasound examination revealed extrahepatic bile duct stones. Thirteen years earlier, she had undergone open cholecystectomy for gallbladder stones. On September 29, 2024, ERCP was performed. Given the large size of the common bile duct stone (approximately 3.9 cm in diameter), direct stone extraction was not feasible. Therefore, EST and biliary plastic stent placement were performed to drain bile and relieve obstruction. Postoperatively, the patient’s abdominal pain and fever subsided.

However, on the 4th postoperative day, the patient developed postprandial abdominal distension and nausea. By the 7th postoperative day, her condition worsened, with fever, vomiting, and difficulty passing stool. Emergency laboratory tests revealed elevated inflammatory markers, bilirubin, pancreatic enzymes and bile enzymes (Laboratory examination indicators are listed in Table 1). CT on October 8 revealed dilatation of the intrahepatic and extrahepatic bile ducts with the presence of pneumobilia, proximal bowel dilation, and high-density shadows of a gallstone and stent within the small intestine, suggesting that the gallstone and stent had migrated into the intestinal tract. These findings were consistent with retrograde biliary infection combined with intestinal obstruction, for which symptomatic medical treatment was administered.

**Table 1:** Laboratory examinations.

Reference range	October 7	October 12	October 15
Neutrophils (0.5–0.7)	0.921	0.626	0.503
White blood cells (3.5 × 10^9^/L–10 × 10^9^/L)	16.37 × 10^9^/L	5.56 × 10^9^/L	5.48 × 10^9^/L
Interleukin-6 (0–5.9 pg/mL)	609.70 pg/mL	5.79 pg/mL	<2.00 pg/mL
Total bilirubin (0–21.0 μmol/L)	57.7 μmol/L	17.5 μmol/L	15.4 μmol/L
Direct bilirubin (0–8.6 μmol/L)	39.5 μmol/L	10.6 μmol/L	10.4 μmol/L
Alkaline phosphatase (0–130 U/L)	653.0 U/L	269.9 U/L	238.7 U/L
Gamma-glutamyl transferase (0–50 U/L)	924.0 U/L	343.9 U/L	296.4 U/L
Amylase (0–150 U/L)	2398.0 U/L	219.1 U/L	212.8 U/L
Lipase (13–60 U/L)	7473.7 U/L	194.0 U/L	373.2 U/L
Blood culture	*Enterococcus faecalis*	–	–

**Figure 1:**
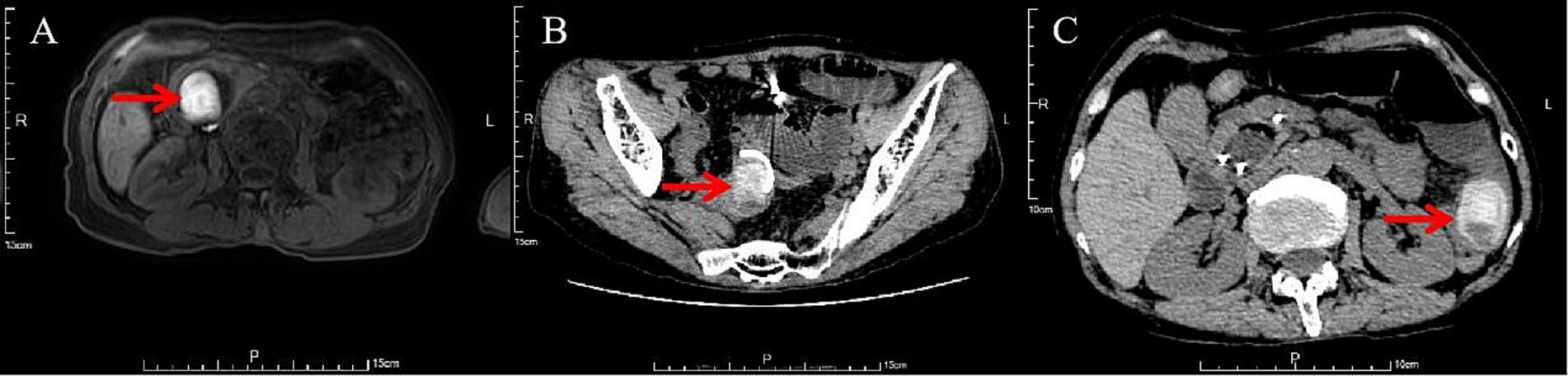
Changes in gallstone location **(A)** October 1 (MRI); **(B)** October 8 (CT); **(C)** October 14 (CT).

On October 11, ERCP was repeated, and biliary and pancreatic stents (for pancreatitis prevention) were placed. Postoperatively, the patient’s abdominal distension and fever improved. A follow-up abdominal CT on October 14 showed that the gallstone had migrated to the 5th–6th segment of the small intestine (the change in the position of the gallstone is shown in Figure 1). After thorough communication with the patient’s family, and considering her poor general condition and comorbid mild tricuspid and aortic valve regurgitation, surgical intervention was deemed high risk and was declined by the patient and her family. As the stone was located near the ileocecal region, endoscopic stone extraction combined with mechanical lithotripsy was considered a feasible option to relieve the intestinal obstruction.

**Figure 2:**
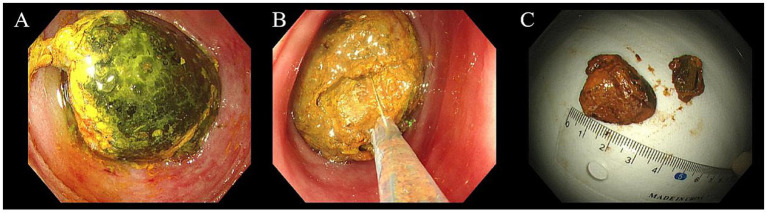
Endoscopic mechanical lithotripsy of gallstone ileus **(A)** large gallstone in the ileum, **(B)** fragmentation of the gallstone, **(C)** retrieved gallstone fragments and size measurement.

On October 16, endoscopic treatment was performed. A colonoscope (OLYMPUS CF-H290I) was advanced through the ileocecal valve to the ileum, 50–70 cm beyond which a large gallstone was found to completely obstruct the intestinal lumen (Figure 2A). Because the outer layer of the gallstone was relatively soft, the stone was fragmented using repeated maneuvers with foreign body forceps, a snare (COOK ASJ-1-S), and a retrieval basket (COOK MWB-3X6) to grasp and pull back portions of the stone (Figure 2B). After the stone size was significantly reduced, the retrieval basket was used to encapsulate and drag the stone to the sigmoid colon. However, owing to the large size of the stone, it could not pass through the sigmoid colon. The scope was advanced to the descending colon, where the stone was completely fragmented using an emergency lithotripter (COOK SLH-1, TTCL-1 [8.5F]), and the larger fragments were extracted using a snare (Figure 2C). The entire procedure lasted approximately 4 hours.

The patient was discharged on October 22. During the 2-month follow-up, no recurrence of symptoms was observed.

## 3 Discussion

According to the literature, it is extremely rare for gallstones to pass directly into the gastrointestinal tract through the ampulla of Vater after ERCP and EST, subsequently leading to gallstone ileus. The onset of this complication can range from 2 days to 7 months post-procedure (3, 6). Most reported cases eventually required surgical intervention; however, there has been one case where the gallstones were spontaneously expelled (6). Although gallstone ileus is an exceedingly rare complication of ERCP, clinicians should remain highly vigilant and closely monitor patients, particularly after the removal of large stones.

Hermosa et al. conducted a retrospective study of 40 patients over 21 years (1980–2000) and reported that the most common site of gallstone impaction in the gastrointestinal tract was the ileum (62.5%), followed by the jejunum (22.5%), duodenum (7.5%), and colon (2.5%). Although the ileum is a frequent site of gallstone ileus, reports of successful endoscopic treatment in this region are limited. Published case reports include Murray et al. (12), who used a large Olympus polypectomy snare to capture and retrieve a gallstone at the ileocecal valve during colonoscopy. De Palma et al. (13) successfully removed an impacted gallstone using a Roth net device. Heinzow et al. (14) was the first to perform single-balloon enteroscopy combined with extracorporeal shock wave, laser, and mechanical lithotripsy to fragment a stone. Shin et al. (15) reported Korea’s first case of a gallstone impacted at the ileocecal valve successfully treated with electrohydraulic lithotripsy.

Endoscopic treatment of gallstone ileus has mostly been reported in case studies, and its applicability should be individually evaluated based on the patient’s condition. Key considerations include the accessibility of the endoscope and instruments, procedural safety, and the patient’s willingness to undergo intervention. In this case, physical examination revealed no significant abdominal tenderness, and the small bowel showed no signs of ischemic necrosis or exudation, suggesting preserved intestinal perfusion. Under these conditions, colonoscopic intervention was considered relatively safe; otherwise, a more cautious approach would be warranted. Additionally, attention should be paid to the patient’s subjective symptoms, as severe complications such as perforation are often accompanied by marked discomfort.

The successful resolution of gallstone ileus may depend on the endoscopist’s selection of instruments and experience level, although these factors have not been systematically evaluated. Endoscopic tools commonly include foreign body forceps, snares, and stone retrieval baskets. Foreign body forceps are typically used to grasp small stone fragments or assist with fragmentation. Snares, although useful, may have issues such as slippage and insufficient gripping strength when dealing with stones. In contrast, stone retrieval baskets are preferred for removing large or irregularly shaped stones. The limited operational space often necessitates combining lithotripsy techniques for completely impacted stones. Laser lithotripsy offers the advantage of minimal trauma in gallstone management (16). However, its probes are prone to damage, and the procedure is expensive. Electrohydraulic lithotripsy is more cost-effective but carries risks such as perforation and bleeding. On the other hand, mechanical lithotripsy is simple to perform, cost-efficient, and has a relatively high success rate when used alone (17, 18). Importantly, most studies on lithotripsy have focused on managing bile duct stones, and sufficient data regarding the removal of gallstones in the intestinal environment are lacking.

A review of the literature identified 24 reported cases of gallstone ileus successfully treated with mechanical lithotripsy (Table 2). The average age of the patients was 78.92 years, with females accounting for 54.17% (13/24) and males accounting for 45.83% (11/24). The duodenum was the most common site of successful cases, accounting for 83.33% (20/24), followed by the colon (12.50%, 3/24) and the jejunum (4.17%, 1/24). The successful application of mechanical lithotripsy in the ileum, as reported in this case, provides an important addition to the data in the table.

**Table 2:** Successful application of mechanical lithotripsy in gallstone ileus.

Gender, age	Number, maximum size (mm), and location of stones	Stone entry route	Endoscopic treatment	Surgical treatment	Year	References
M, 71	1, NA, bulb	D1 has a large fistulous opening	–	Planned cholecystectomy	2016	Dumonceau and Deviere (18)
F, 83	1, NA, bulb	Cholecystoduodenal fistula	–	–	2015	
M, 78	1, NA, pylorus	Cholecystoenteric communication	–	–	2015	
M, 90	1, NA, pylorus	Cholecystoduodenal fistula	Endoscopic biliary sphincterotomy, common bile duct stone extraction	–	2014	
F, 62	1, 35 × 30, D3	Cholecystoduodenal fistula	–	–	2012	
F, 81	1, 30, bulb	Cholecystoduodenal fistula	–	–	2012	
M, 84	1, 40, pylorus	Bilio-enteric fistula	–	Planned cholecystectomy	2012	
M, 80	1, 45 × 40, bulb	NA	–	–	2011	
F, 81	2, NA, pylorus	Cholecystoduodenal fistula		Extended cholecystectomy (incidental T1 adenocarcinoma)	2010	
M, 86	1, 33, D2	Cholecystoduodenal fistula	–	–	2009	
F, 93	1, 27 × 27, bulb	Cholecystoduodenal fistula	–	–	2009	
F, 76	1, NA, bulb	Cholecystoduodenal fistula	–	–	2008	
F, 76	1, 30, bulb	Possibility of cholecystoduodenal fistula	–	–	2008	
F, 65	1, 35 × 25, pylorus	Cholecystoduodenal fistula	–	–	2007	
M, 81	1, NA, bulb	Possibility of cholecystoduodenal fistula	–	–	2006	
F, 73	1, 40, bulb	Cholecystoduodenal fistula	–	Cholecystectomy, closure of the cholecystoduodenal fistula	2005	
M, 68	1, 25, bulb	NA	–	Cholecystectomy	2002	
M, 86	1, 25 × 30, bulb	Choledochoduodenal fistula	–	Cholecystectomy	2017	Hasan et al. (19)
F, 83	1, 35, D1	Cholecystoduodenal fistula	–	–	2022	Sanyang et al. (20)
M, 61	1, 50, duodenum	The fistula was observed along the lateral duodenal wall	–	Planned outpatient cholecystectomy	2023	Bechara et al. (21)
F, 94	1, 35, sigmoid colon	Cholecystocolonic fistula	–	–	2015	Balzarini et al. (22)
F, 78	1, 30, sigmoid colon	NA	–	–	2008	Reiss et al. (23)
M, 73	1, 40 × 40, rectosigmoid level	Cholecystoenteric fistula	–	–	2014	Waterland et al. (24)
F, 91	1, 40, upper jejunum	Cholecystoduodenal fistula	–	–	1999	Lubbers et al. (25)

M, male; F, female; NA, not available; D1, first part of the duodenum; D2, second part of the duodenum; D3, third part of the duodenum.

Based on our procedural experience, we recommend first assessing the size of the gallstone to determine whether it can be directly retrieved. If the stone is large, its volume should be reduced *in situ* before attempting extraction. When sufficient operating space is available, a snare and retrieval basket can effectively fragment and remove the stone. However, if the stone is tightly adherent to the intestinal wall, preventing proper deployment of these devices, initial fragmentation using foreign body forceps may help create adequate space for subsequent instrument access and lithotripsy.

## 4 Conclusion

In gallstone ileus, stones are often impacted, significantly increasing the difficulty of treatment with a single instrument. In this case, multiple tools, including foreign body forceps, snares, stone retrieval baskets, and emergency lithotripters, were used to fragment and completely remove the stone gradually. This highlights the value of a multitool approach in managing complex cases.

## Data Availability

The original contributions presented in the study are included in the article/supplementary material, further inquiries can be directed to the corresponding authors.
